# Is High Fat and Sugar Intake Associated with Disrupted Attentional–Motivational Coupling for Food? Evidence from an Eye Tracking Study

**DOI:** 10.3390/brainsci16060648

**Published:** 2026-06-18

**Authors:** Tuki Attuquayefio, Olivia Lauren Aguiar, Bandal Boutros, Peter Jacquier, Richard J. Stevenson, Gesualdo M. Zucco

**Affiliations:** 1 School of Psychology, Faculty of Health Sciences, Western Sydney University, Sydney 2751, Australia20735441@student.westernsydney.edu.au (O.L.A.); 20623236@student.westernsydney.edu.au (P.J.); 2 School of Psychological Sciences, Macquarie University, Sydney 2109, Australia; 3 School of Medicine, Department of Philosophy, Sociology, Education and Applied Psychology, University of Padova, 35139 Padova, Italy

**Keywords:** high fat, high sugar, discretionary intake, Western diet, eye tracking, eating behaviour, overeating, visual attention, bogus taste test, snacking, appetite, satiety

## Abstract

**Background**: Frequent consumption of foods high in fat and sugar (HFS) has been linked to disrupted appetite regulation (via hippocampal dysfunction) and an increased tendency to continue desiring palatable foods, even when physiologically full. While we have previously shown that motivational drive for such foods can persist when full, it remains unclear whether attentional engagement (i.e., the visual attention captured by palatable foods) shows a similar sustained desire to consume palatable foods when full. Understanding whether attention persists is critical, as attention can powerfully shape food choice and overeating. **Methods**: This study investigates whether habitual HFS intake was associated with the maintenance of visual attention, motivational responses, and food consumption when satiated. Twenty-four adults aged 18–30 years completed a food frequency questionnaire and a bogus taste-rating task once when hungry and again after consuming a standardised meal. Using Tobii Pro Glasses 3 wireless eye-tracking glasses, we measured fixations on real snack foods, and participants rated wanting and liking for each item. **Results**: Eating a meal significantly reduced total fixations to snack foods, and wanting was more sensitive than liking to physiological state. Fixations were higher for ‘healthy’ snacks compared to ‘unhealthy’ snacks, with this effect more pronounced when participants were hungry. Notably, individuals in the low-fat/low-sugar group showed strong alignment between post-meal decreases in visual attention and decreases in wanting and liking, whereas this coupling was diminished in the high-fat/high-sugar group. **Discussion**: Extending previous work into the domain of attention, this study reveals diet-related differences in how visual attention interacts with motivational evaluations of food. The disrupted coupling associated with high-fat/high-sugar intake suggests potential alterations in attentional and motivational processes supporting appetite regulation. Understanding how diet shapes these cognitive–motivational interactions provides a valuable foundation for future neurocognitive research on overeating and obesity risk.

## 1. Introduction

Obesity, type 2 diabetes, and dementia place a growing burden on health systems and quality of life, and dietary composition is a key modifiable factor implicated in these conditions [1,2]. A common pattern in industrialised contexts is the frequent dietary intake of foods high in saturated fat and refined sugar (hereafter referred to as ‘HFS’ foods), and consumption of these foods is associated with metabolic dysfunction and adverse neurocognitive outcomes [2]. Food environments that normalise energy-dense, hyper-palatable products encourage intake beyond metabolic need, and these products are reliably linked to higher ad libitum energy consumption across dietary patterns, indicating a direct behavioural route by which such foods promote overconsumption [3].

### 1.1. HFS Foods, Satiety, and Wanting

Under normal conditions, people should stop eating when they are full. One model argues that satiety signals are processed through the hippocampus to reduce appetitive responses to food cues [4]. As such, under normal conditions, the wanting and liking of foods are modulated by physiological state, increasing with hunger and decreasing with satiety. However, there is emerging evidence in humans that habitual HFS intake impairs hippocampal function and integrity, and this diet-induced hippocampal dysfunction weakens the use of interoceptive signals to stop eating, resulting in overeating that leads to further dysfunction [1]. A wealth of animal evidence supports this model [5], and there is emerging support from humans that HFS foods negatively affect hippocampal processes [6,7,8,9,10].

In humans, observational and experimental work suggests that greater consumption of HFS foods undermines hippocampal function. In a cross-sectional human study, higher habitual HFS intake is associated with poorer performance on hippocampal-related memory tasks [6,9,11] and with a reduced ability to suppress wanting for palatable foods after eating [6,9]. In another study, wanting ratings decreased more than liking for solid snacks, but wanting reduced minimally for sugar-sweetened beverages. Specifically, after consuming energy-replenishing beverages, individuals with higher habitual intake of high-fat foods reported stronger wanting for sweet stimuli while liking returned toward baseline, indicating that motivational drive can remain elevated when hedonic valuation normalises following repletion [10].

Across human and animal studies, even brief exposure to HFS foods can impair learning and memory functions that support the self-regulation of eating, suggesting that diet quality influences cognition and eating behaviour on rapid timescales as well as over longer periods [5,6]. Multiple experimental studies have also shown that shifting healthy adults to a diet with HFS foods produced measurable reductions in hippocampal-dependent learning and interoceptive sensitivity, showing that short-term diet manipulation can alter mechanisms that help terminate consumption [7]. According to Incentive Salience Theory, ‘wanting’ drives automatic capture towards food cues [12]. This aligns with the idea that attention is motivated by reward salience rather than just visual interest. However, the relationship between habitual HFS intake and visual attention to foods remains unexplored.

### 1.2. Visual Attention

Eye-tracking methodologies have examined visual attention toward food cues. Visual attention includes eye-tracking indices such as fixation duration, dwell time, and gaze frequency, which quantify this process in real time [13]. Studies suggest that visual attention toward food varies with satiety state, reward sensitivity, and dietary history, indicating that both internal and learned factors shape the degree to which cues dominate perception [13,14]. For instance, overweight individuals showed increased initial orientation and maintained gaze on calorie-dense food compared to lean controls [15]. Similarly, individuals with higher Body Mass Index (BMI) showed greater attentional engagement with food-related stimuli, particularly when hungry [16]. Likewise, using eye-tracking, individuals with obesity maintained longer visual attention to palatable food images compared to lean controls [17]. In controlled tasks, individuals with binge-eating tendencies maintained heightened gaze on high-calorie foods when satiated compared with controls, supporting the view that attention to palatable cues can remain elevated independent of immediate energy needs [13]. Collectively, these studies suggest that visual attention toward palatable foods is more robust in populations vulnerable to overeating. While eye tracking has previously been used to investigate attention to food cues [15,16], few studies have examined the differences in visual attention based on habitual diet, food palatability, and physiological state.

### 1.3. Aims

The present study examines whether individuals with higher habitual intake of HFS foods differ from those with lower fat and sugar (LFS) intake in their visual attention to foods, motivational responses (i.e., ratings of wanting and liking for snack foods), and subsequent eating behaviour across hunger and satiation. The present study addresses two critical questions: (1) whether attentional processes are also impaired by habitual HFS intake, and (2) whether visual attention is specific to unhealthy food cues or reflects a generalised attention toward all foods among HFS consumers when satiated. Importantly, this study tests not only whether these processes differ, but whether their functional coupling is preserved or disrupted as a function of habitual diet.

### 1.4. Hypotheses

Based on the literature examined above, we hypothesised that following a meal, the HFS group would show:

**H1.**  *Smaller changes in wanting but not liking all snacks compared to the LFS group.*

**H2.**  *More fixations on all snacks compared to the LFS group.*

**H3.**  *Higher fixations on unhealthy foods compared to the LFS group.*

**H4.**  *More fixations are linked with greater snack intake, and this association would be stronger for unhealthy snacks.*

### 1.5. Novelty and Contribution

Critically, although prior research has independently demonstrated that habitual high fat and sugar (HFS) intake affects motivational responses to food and that visual attention to food varies with physiological and individual differences, these domains have largely been investigated in isolation. The present study addresses this gap by directly examining the coupling between attentional allocation (fixations) and motivational value (wanting and liking) as a function of habitual diet. By combining eye-tracking with concurrent measures of motivation and behaviour in an ecologically valid setting using real foods, the current study provides a novel test of whether attentional processes track motivational relevance differently depending on dietary phenotype. This approach extends existing behavioural findings into a mechanistic attentional domain, offering new insight into how habitual diet may alter the integration of attention and motivation during eating.

## 2. Materials and Methods

### 2.1. Participants

Participants included adults who were recruited through the Western Sydney University SONA research participation system and community advertisements. Participants were included if they reported no current dieting, diagnosed eating disorders, metabolic conditions, or uncorrected vision impairments that could influence eating behaviour or eye-tracking accuracy. Our previous work shows large effects of diet on behavioural outcomes [7]. To adopt a conservative approach, the present study was powered to detect medium-sized effects, rather than relying on previously reported large effects. An a priori power analysis conducted in G*Power (Version 3.1) [18] indicated that a total sample of 24 participants would provide adequate statistical power (1 − β = 0.80, α = 0.05) to detect a medium effect size (η^2^ = 0.07) in a mixed design with one between-subjects and two within-subjects factors. This approach reflects standard practice in experimental psychology, where effect sizes are conservatively estimated to avoid overestimating statistical sensitivity. Additionally, the repeated-measures design and use of continuous eye-tracking metrics increase statistical efficiency relative to purely between-subjects behavioural paradigms.

### 2.2. Materials

#### 2.2.1. Dietary Fat and Sugar Questionnaire (DFS) [19]

The DFS is a 26-item self-report measure assessing habitual consumption of HFS foods. Food items (e.g., sausages, chocolate) are rated on a 5-point Likert scale ranging from 1 (rarely or never) to 5 (once a day or more). Total scores range from 26 to 130, with higher scores reflecting greater habitual intake of HFS foods. The scale has demonstrated excellent internal reliability (α = 0.85). Participants were grouped using a median split based on total DFS scores (*Mdn* = 61); <61 as the LFS diet group and ≥61 as the HFS diet group. (12 per group). This approach was adopted to facilitate comparison with prior work in this area that has used similar group-based classifications [6,9] and to align with the study’s focus on between-group differences in behavioural and attentional responses.

#### 2.2.2. Food Stimuli

A total of 8 different foods were used as stimuli for a taste test (outlined below). Food stimuli comprised two broad categories: ‘unhealthy’ and ‘healthy’ foods. Unhealthy items were selected to reflect the definition and perception of hyperpalatable foods, high in fat and sugar [20]. Four ‘unhealthy’ snack foods included: chocolate chip cookies (Coles Group Pty Ltd., Melbourne, VIC, Australia), Smith’s salt and vinegar chips (The Smith’s Snackfood Company Pty Ltd., North Sydney, NSW, Australia), Doritos (Frito-Lay Inc., Plano, TX, USA) plain salted corn chips, and M&M’s chocolates (Mars Inc., McLean, VA, USA). Healthy items consisted of more nutritious, less energy-dense foods lower in fat and sugar levels that align more with ‘healthy’ foods. The four ‘healthy’ items included: untoasted almonds (Coles Group Pty Ltd., Melbourne, VIC, Australia), raisins (Coles Group Pty Ltd., Melbourne, VIC, Australia), Bega (Bega Group Pty Ltd., Bega, NSW, Australia) cheese cubes, and dried banana chips (Coles Group Pty Ltd., Melbourne, VIC, Australia). All foods were pre-weighed and portioned into visually equal serving sizes (50 g for all snacks except chips and Doritos, which were 30 g due to lighter weight).

#### 2.2.3. Bogus Taste Test (BTT)

**Figure 1:**
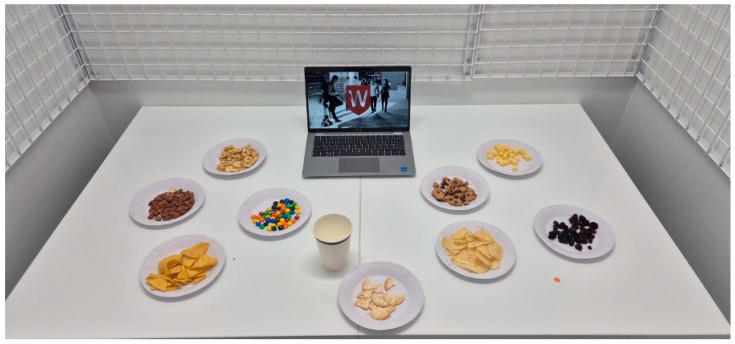
Bogus taste test setup displaying the eight food stimuli (four unhealthy and four healthy) presented during the eye-tracking and BTT tasks.

Food intake was measured using the bogus taste test (BTT), a validated laboratory measure of eating behaviour [21]. The BTT was used as a covert behavioural measure of food intake. The BTT provides an ecologically valid and widely used measure of spontaneous eating behaviour under controlled conditions and correlates with real-world ad libitum intake [6,9,21]. Participants were presented with pre-weighed bowls of 4 healthy snacks and 4 unhealthy snacks in front of them on a table setup (see Figure 1) under the guise of evaluating taste characteristics (sweetness, saltiness, crunchiness, healthiness, fattiness). Participants sampled each food item individually and were asked to taste as much as needed to form their evaluations. Participants rated each food immediately after viewing or tasting it. A cup of water and a bowl of plain wafer cracker pieces were provided as a palate cleanser between each snack tasting. The order of snacks was randomised each time the BTT was completed and across participants.

#### 2.2.4. Appetite Ratings

Participants completed Visual Analogue Scale (VAS) ratings of hunger (“How hungry do you feel right now?”), fullness (“How full do you feel right now?”), and thirst (“How thirsty do you feel right now?”), anchored from 0 (not at all) to 100 (extremely).

#### 2.2.5. Wanting and Liking Ratings

Subjective motivational responses were assessed using VAS for wanting (“How much do you want to eat this food right now?”) and liking (“How pleasant does this food taste?”). Wanting was defined as the desire to consume food upon visual presentation, while liking reflected the hedonic enjoyment upon tasting [12].

#### 2.2.6. Eye Tracking

Eye-tracking data were collected using Tobii Pro Glasses 3, a lightweight, glasses-mounted eye-tracking system that allows free viewing of real-world stimuli. The glasses were connected to a recording unit and a laptop for live monitoring via the Tobii recording software. The sampling rate was 50 Hz. Calibration was completed at the start of each session using a single-point fixation target presented at arm’s length as per the manufacturer’s instructions. Fixations were defined according to Tobii’s default settings as a minimum of 60 ms gaze to a single point, with a maximum allowed between fixations of 0.5 degrees and 75 ms. We used Tobii’s homography-based assisted mapping algorithm (called ‘Real World Mapping’) to map areas of interest for snacks in the video output against a high-resolution image of the BTT setup (Figure 1), with the confidence level set at 90%. Any inaccuracies in assisted mapping between the standard still image and the eye-tracking video output were corrected manually by the authors. Two key metrics were derived from the eye-tracking data and extracted using the Tobii Lab Analyser software (version 24.21): total fixation duration and fixation count. Total fixation duration represents the cumulative time spent focusing on a specific area of interest (i.e., the snacks). Fixation count was defined as the total number of discrete fixations directed toward the food stimuli. Notably, eye-tracking measures such as fixation duration and count provide high-resolution, continuous indices of attentional allocation, which can increase sensitivity relative to discrete behavioural outcomes. These metrics provide objective measures of overt attention, reflecting both initial orienting and sustained engagement with stimuli [13].

### 2.3. Procedure

This study adopts a mixed-group observational design. Each participant attended a single, individual laboratory session lasting approximately 45 min. Testing was conducted in a quiet, temperature-controlled laboratory at Western Sydney University to minimise distraction and standardise environmental conditions. Participants were seated at a testing table approximately 60 cm from a 15-inch laptop screen used to display task instructions and VASs. Participants were instructed to fast for 10 h prior to the test. On arrival, fasting was confirmed and consent obtained. They then completed demographic questions and the DFS to determine dietary classification. Participants completed the BTT over two phases—hungry and satiated—within the same lab session. In each phase, participants completed the BTT under the cover story that it was a separate study on taste perception. They were told to try and rate each snack food and could eat freely during the 10 min task. Food intake was covertly measured by comparing the pre- and post-task weights of all snacks. To encourage consumption, they were informed that all food would be discarded and were given 10 min to eat as much as they would like. Snack bowls were then removed. Participants provided appetite ratings, then consumed a standardised lunch (sandwich 1450 kJ, granola bar 830 kJ). Ten minutes after finishing their meal, appetite ratings were again taken, followed by the BTT another 5 min later (15 min post-meal). Participants were debriefed with the true purpose of the study and the use of the cover story for the BTT. Participants were thanked for their participation and awarded $20 or SONA credit points.

### 2.4. Analysis

Analyses were structured into primary (confirmatory) and secondary (exploratory) components. Primary analyses consisted of planned factorial ANOVAs aligned with the study hypotheses. Exploratory analyses included correlation analyses and between-group comparisons of correlations, for which false discovery rate (FDR) correction was applied within families of tests to control for multiple comparisons.

For primary analyses, the between-subjects factor was the habitual diet group (HFS vs. LFS), determined using the DFS. The within-subject factors were physiological state (hungry vs. sated) and snack type (unhealthy vs. healthy). The key dependent variables were total fixation and fixation count. Wanting and liking ratings obtained via VAS and grams of food consumed in each round of the BTT were also included to examine subjective motivation and behavioural intake. One participant showed extreme outlier data points (*z* > ±3.29) [22] across six measures, and their data were removed from relevant analyses. Assumptions of sphericity, normality, and heterogeneity of variance were met. A 3-way mixed ANOVA was used to examine the interaction between habitual diet group (HFS vs. LFS), physiological state (hungry vs. sated), and snack type (unhealthy vs. healthy). For any significant interactions, simple effects analyses and pairwise comparisons were Bonferroni-adjusted.

Exploratory analyses included correlation analyses between attentional and motivational measures, as well as between-group comparisons of correlation coefficients (Fisher’s Z tests). These analyses involve multiple related tests and were therefore corrected using a false discovery rate (FDR) procedure to limit the expected proportion of false positives within these families of tests.

## 3. Results

The final sample was 24 participants (*M* = 22.58, *SD* = 2.73, 10 males, 14 females), BMI (*M* = 26.37, *SD* = 5.03), and diet group (HFS = 12, LFS = 12). Descriptive statistics and standard deviations for each diet group are presented in Table 1. Importantly, the groups did not differ substantially in BMI or key demographic variables (Table 1), reducing (but not eliminating) the likelihood that these factors account for the observed effects.

### 3.1. Snack Intake

**Figure 2:**
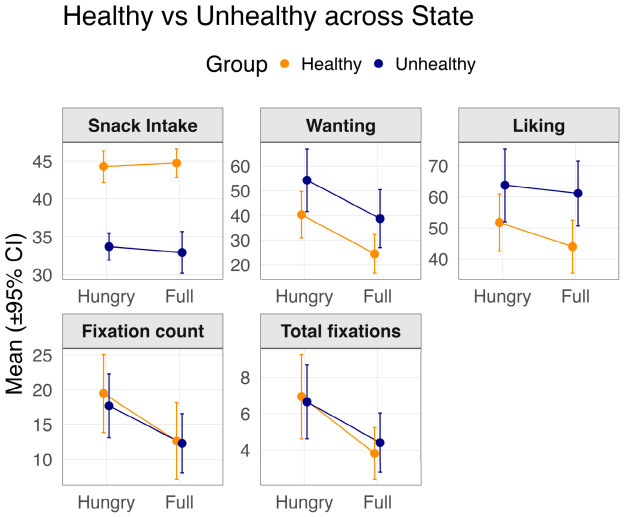
Line plots of key measures between hungry and full, for healthy and unhealthy snacks.

Snack intake was measured as grams consumed during the BTT. A 2 (State: Hungry, Sated) × 2 (Diet Group: HFS, LFS) × 2 (Snack type: Healthy, Unhealthy) mixed ANOVA was conducted on total grams consumed. The full results are reported in Table A1. There were no main effects for diet group (HFS vs. LFS) or state (hungry vs. full), but there was a main effect of snack type, *F*(1,21) = 217.72, *p* < 0.01, η^2^ = 0.91. Specifically, participants consumed significantly more ‘healthy’ foods (*M* = 44.52, *SD* = 4.69) compared to ‘unhealthy’ foods (*M* = 33.32, *SD* = 5.44). Figure 2 shows the main effect of snack type on the weight of snacks consumed. No other interactions between diet group, state, and snack type were observed for snack intake. Interestingly, when participants were hungry, snack intake was strongly negatively related to fixation count, *r*(21) = −0.750, *p* < −0.001, and total fixations, *r*(21) = −0.784, *p* < 0.001. When full, snack intake was still strongly related (though less so) with fixation count, *r*(21) = −0.521, *p* = 0.015, and total fixations, *r*(21) = −0.469, *p* = 0.003.

### 3.2. Subjective Wanting and Liking Ratings

For both wanting and liking ratings, there were main effects of snack type and state (see Table A2). No differences based on diet group were found, and no other interactions were observed. In line with previous findings [6,9], we found a significant interaction between state and rating type. Specifically, wanting ratings decreased significantly more between hungry (*M* = 47.33, *SD* = 27.20) and full (*M* = 31.64, *SD* = 24.55) compared to decreases in liking ratings when hungry (*M* = 57.78, *SD* = 25.45) and full (*M* = 52.54, *SD* = 23.94; *F*(1,22) = 11.93, *p* = 0.002, η^2^ = 0.352). Additionally, there was a trend showing that satiation-related differences between wanting and liking ratings were different based on diet group, *F*(1,22) = 3.18, *p* = 0.09, η^2^ = 0.126.

### 3.3. Visual Attention for Snack Foods

**Figure 3:**
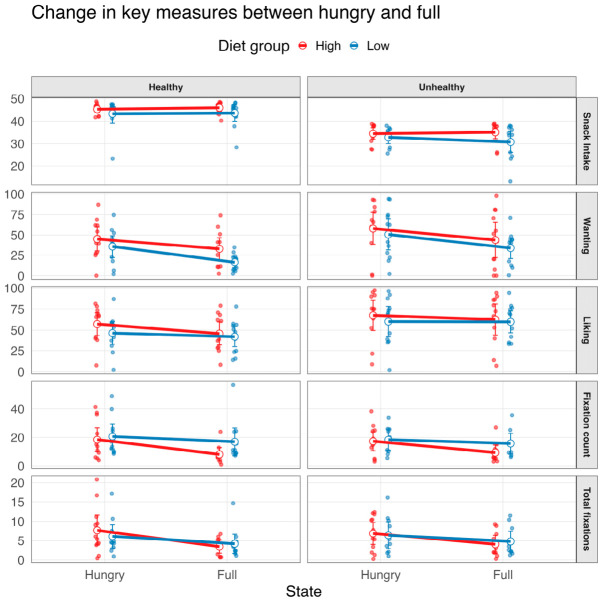
Line plots of key measures by diet group and between hungry and full, for healthy and unhealthy snacks.

Visual attention was examined using two key variables: total fixation and fixation counts. For fixation count, there was a significant main effect of snack type, *F*(1,17) = 6.41, *p* = 0.02, η^2^ = 0.27), with significantly more fixation counts to healthy snacks (*M* = 16.22, *SD* = 12.85) compared to unhealthy snacks (*M* = 15.13, *SD* = 9.75). There were no differences in fixation count based on physiological state or diet group, and no other interactions were found (see Table A1 for full model outputs). For total fixations, there was a significant main effect of state, *F*(1,18) = 9.66, *p* = 0.01, η^2^ = 0.35), with significantly more total time spent fixating on food (regardless of snack type or state) (*M* = 6.80, *SD* = 4.95) compared to when full (*M* = 4.10, *SD* = 3.28). This finding is in line with previous findings [6,9,17], with a reduction in visual attention when participants are satiated. No other main effects or interactions were found, including no differences based on diet group. The full results are presented in Table A1 and plotted across all measures in Figure 3.

### 3.4. Exploratory Analyses

**Figure 4:**
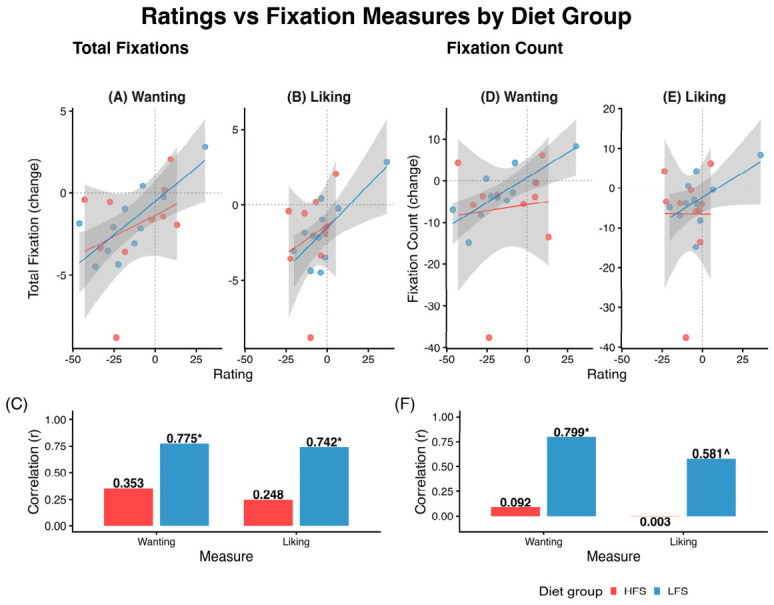
Scatterplots of total fixations by diet group for change in (**A**) wanting and (**B**) liking. (**C**) Comparison of correlation coefficients for total fixations and each rating by diet group. Scatterplots of fixation count by diet group for change in (**D**) wanting and (**E**) liking. (**F**) Comparison of correlation coefficients for fixation count and each rating by diet group. * *p* < 0.05; ^ *p* < 0.10.

Based on our previous findings [6,9], we explored additional relationships between our key variables. Interestingly, the LFS group showed significant positive correlations between total fixations and both liking, *r*(11) = 0.74, *p* = 0.02, and wanting ratings, *r*(11) = 0.78, *p* = 0.02. The HFS did not show correlations between total fixations and liking, *r*(10) = 0.25, *p* = 0.49, or wanting, *r*(10) = 0.35, *p* = 0.42. These correlations maintained the same patterns of significance following false discovery rate (FDR) correction for multiple comparisons (FDR-corrected values reported here). Fisher’s Z-tests of diet group differences between correlations were not significant for total fixation-liking, *Z* = −1.28, *p* = 0.199, and for total fixation-wanting, *Z* = −1.36, *p* = 0.175; see Figure 4C.

For the fixation count, we observed similar results. The LFS group showed strong correlations between changes in fixation count and wanting, *r*(11) = 0.80, *p* = 0.012, but not for liking, *r*(11) = 0.58, *p* = 0.12, while the HFS did not show these relationships with wanting, *r*(10) = 0.09, *p* = 0.99, and liking, *r*(10) = −0.003, *p* = 0.99; see Figure 4D,E. These correlations maintained the same patterns of significance following FDR correction for multiple comparisons. Fisher’s Z-tests of diet group differences between correlations showed a trend for total fixation-liking, *Z* = −1.94, *p* = 0.052, and was non-significant for total fixation-wanting, *Z* = −1.29, *p* = 0.197; see Figure 4F.

## 4. Discussion

This study aims to examine whether habitual HFS intake was associated with greater visual attention to foods when full. The present study provides the first direct test of whether attentional allocation to food cues is functionally coupled with motivational value as a function of habitual diet. While we did not observe robust group differences in mean levels of attention or intake, the key contribution lies in the observation that the relationship between visual attention and motivational evaluations differed systematically across diet groups, suggesting a diet-related alteration in how attentional and motivational systems interact.

The first hypothesis predicted that, after a meal, the HFS group would show smaller changes in wanting but not liking for all snacks compared to the LFS group. We did not find a significant interaction between diet group, rating type, and state (noting that there was a trend to significance). Despite this, subjective motivation showed clear state sensitivity: feeling full reduced wanting much more than liking, replicating the well-established dissociation in which satiety dampens motivational drive while hedonic evaluation remains relatively intact. There was also a suggestive pattern (trend) that this satiety-related reduction in wanting was weaker in the HFS group, consistent with previous work indicating impaired motivational regulation in individuals consuming an HFS diet [6,9,10]. Both groups experienced declines in wanting and liking after satiety, but a trend indicated the reduction in wanting was less pronounced in the HFS group. This trend aligns with the IST [12], which proposes that repeated exposure to highly palatable foods enhances cue-triggered motivation (“wanting”) independently of subjective pleasure (“liking”). The current findings suggest that habitual HFS consumption may modestly blunt the inhibitory effect of satiety on food motivation.

The second hypothesis proposed that participants in the HFS group would display greater visual attention toward food stimuli during the BTT compared to the LFS group. This was not supported, as fixation counts and total fixation duration did not differ between diet groups.

The third hypothesis predicted fewer fixations on unhealthy foods after a meal for the LFS group, while the HFS diet group would maintain high fixation. Overall, participants demonstrated higher fixation counts for healthy foods compared to unhealthy foods, and state-based changes in total fixation duration. Ultimately, both these findings were not different based on the diet group.

The fourth hypothesis stated that snack intake would be related to visual attention. Interestingly, visual attention showed complementary effects, with participants spending more time looking at food when hungry and directing slightly more fixations toward healthy snacks, indicating that attentional engagement with food is sensitive to physiological state and stimulus characteristics. This finding still links attentional engagement to behavioural outcomes in a way that supports a causal role for attention in satiety-resistant eating [23]. Unexpectedly, we found evidence of a relationship between snack intake and visual attention, though in the opposite direction than predicted. Greater visual attention to all snacks was associated with consuming less snack food. Participants who paid more attention to the food stimuli consumed less, suggesting that sustained visual engagement may support, rather than hinder, self-regulatory control overeating. Likewise, lower intake of unhealthy snacks may suggest active inhibition of intake specifically for unhealthy snacks, and this process may drive attentional metrics. Alternatively, this finding may be an unintended consequence of the BTT, where we ask participants to focus on the characteristics of the snacks, and these task demands may constrain naturalistic eating behaviours.

In our exploratory analyses, we discovered something interesting in the interactions and between-groups correlations (noting here this was exploratory and sample size constraints). The pattern of results suggested a diet-based coupling between motivational responses and visual attention. That is, the relationship between attention and subjective value differed by diet group: in the LFS group, reductions in attention when full were closely aligned with reductions in wanting, reflecting an intact coupling between motivational value and attentional allocation. In contrast, the HFS group showed a marked decoupling of attention from wanting and liking, with state-based changes in visual attention no longer tracking with changes in subjective motivation. While the results did not reach significance (possibly due to sample size), this pattern is suggestive, but not directly demonstrated, and consistent with prior work showing that HFS impairs the ability to reduce wanting when full [6,9]. While more research is needed, these findings suggest that habitual HFS consumption is associated with an impairment in the integration of satiety, motivation, and attention, potentially undermining adaptive reductions in food-related motivation and attentional engagement when energy needs are met. This finding advances prior work by demonstrating that the impact of habitual diet may lie not in isolated changes in attention or motivation, but in the decoupling of these processes, representing a previously untested mechanism linking diet to dysregulated eating behaviour.

### Future Directions

The current study shows that wireless eye-tracking metrics can be used with real snack foods to track both visual attention, consumption behaviours, and motivational responses. This work addresses a gap in the literature by integrating motivational and attentional processes with direct behavioural measures in an ecologically valid setting [1,12,13]. Using real foods and simultaneous measurement of gaze and intake provides an ecologically relevant context for testing whether habitual HFS intake is associated with sustained attention to palatable foods when full and with greater consumption in the same setting [23,24].

Wireless eye-tracking technology was used to measure visual attention to real food, and intake was covertly recorded using the taste test paradigm [3,9]. This methodological combination strengthens ecological validity by using real foods rather than static images and by linking attentional patterns directly to behavioural intake in the same setting [23,24]. The eye-tracking data used fixation count and total fixation duration as the key metrics of visual attention. Fixation count, while objective, is a broad measure that does not capture the temporal dynamics of attention. Metrics such as dwell time or first fixation latency could reveal more nuanced attentional differences. Future studies should incorporate additional attention metrics, such as dwell time and initial orienting, to capture more nuanced attention behaviours.

While the study was adequately powered to detect medium-sized effects, it may have been underpowered to detect smaller or more subtle diet-related differences, particularly given the modest separation between groups resulting from the median split. With N = 24 (12 per group), the design had adequate sensitivity only for large interaction effects (*f* = 0.25); smaller effects should be interpreted cautiously. As such, the absence of significant group effects should be interpreted with caution, and future studies with larger samples or more extreme group stratification may be better positioned to detect finer-grained effects. Given the sample size and scope of analyses, the study was not designed to provide a fully controlled family-wise error rate across all tests. The sample consisted mostly of lean-weight individuals whose dietary habits were not particularly extreme (range was 40–88, but potential scores could range from 26 to 130). This range of HFS intake may have limited the detection of diet effects on visual attention, though residual confounding cannot be ruled out and should be considered in future research. Moreover, future studies should match ‘healthy’ and ‘unhealthy’ snacks on multiple attributes, such as energy density and palatability, to control for these factors.

Across all measures, we did not find group differences between diet groups. This is most likely an artefact of using a median split to determine groups, rather than using upper and lower tertiles to form groups, as in previous studies [6,9]. Although this approach was useful for comparing participants with relatively higher and lower intake of HFS foods, it reduces statistical power and sensitivity by artificially dichotomising a continuous measure [25]. Consequently, the difference between HFS and LFS groups is likely modest, which may have obscured finer-grained relationships between habitual diet and cognitive or behavioural outcomes. Future studies with larger samples should model DFS continuously to provide a more precise test of the underlying theoretical framework.

## 5. Conclusions

This study examines whether habitual HFS consumption was related to cognitive and behavioural markers of satiety resistance. Overall, these results highlight the complexity of attentional processes based on physiological state and habitual diet. Visual attention was related to snack intake, though no diet-related differences were noted. The absence of group differences in mean attentional allocation suggests that habitual diet may not influence how much attention is allocated to food stimuli, but rather how that attention relates to motivational value, highlighting the importance of examining interactions between systems rather than main effects alone. In line with our previous work, wanting and liking ratings showed diet-based differences. This study adds visual attention evidence to this body of work. Specifically, we show that lower fat and sugar intake was associated with appropriate coupling of visual attention and motivational processes, while higher fat and sugar intake does not show this coupling, suggestive of a disruption in processes related to appetite regulation, motivation, and attention. These findings underscore the importance of understanding how habitual diet and physiological state jointly influence the complex attentional mechanisms that operate in everyday food environments. Future research employing larger, well-powered samples, refined attentional and behavioural measures, and neurobiological indices of satiety will be essential for clarifying the mechanisms linking habitual HFS consumption to eating beyond physiological need.

## Figures and Tables

**Table 1:** Sample descriptives.

Variable	HFS Group (*n* = 12)	LFS Group (*n* = 12)	*p*-Value
Age	23.00 (3.05)	22.17 (2.44)	0.519
BMI	26.36 (4.49)	26.38 (5.72)	0.887
Sex			>0.999
Female	7 (58%)	7 (58%)	
Male	5 (42%)	5 (42%)	
Race			0.819
Asian	3 (25%)	3 (25%)	
Caucasian	1 (8.3%)	2 (17%)	
Other	8 (67%)	7 (58%)	
Wanting (hungry)	51.52 (25.01)	43.15 (23.17)	0.514
Wanting (full)	38.27 (24.10)	25.00 (14.10)	0.143
Liking (hungry)	62.31 (21.90)	53.24 (22.54)	0.298
Liking (full)	54.13 (20.79)	50.95 (17.08)	0.590
Snack Intake (hungry)	39.89 (2.95)	37.09 (6.98)	0.273
Snack Intake (full)	40.52 (3.39)	37.18 (6.02)	0.088
Total Fixations (hungry)	7.29 (5.08)	7.10 (5.84)	0.740
Total Fixations (full)	3.73 (2.68)	5.33 (5.13)	0.426
Fixation Count (hungry)	17.79 (11.58)	25.80 (21.15)	0.424
Fixation Count (full)	8.68 (6.68)	22.85 (21.67)	0.020 *

^1^ Mean (SD); n (%) ^2^ Wilcoxon rank sum test; Wilcoxon rank sum exact test; Pearson’s Chi-squared test. * *p* < 0.05.

## Data Availability

The raw data supporting the conclusions of this article will be made available by the authors on request.

## Abbreviations

The following abbreviations are used in this manuscript:

HFS	High fat and sugar
LFS	Low fat and sugar
DFS	Dietary Fat and Sugar Questionnaire
BTT	Bogus Taste Test
VAS	Visual Analogue Scale
ANOVA	Analysis of Variance

## Appendix A

**Table A1:** Output of 3-way (State (hungry vs. full) × Snack type (healthy vs. unhealthy) × Diet group (HFS group vs. LFS group) ANOVA models for each measure. * *p* < 0.05.

Measure	Effect	DFn	DFd	SSn	SSd	F	*p*-Value	Partial Eta Squared
Snack intake	Diet group	1	21	58.83	920.481	1.342	0.26	0.06
Snack intake	Snack type	1	21	3214.808	310.081	217.721	<0.001 *	0.912
Snack intake	State	1	21	0.108	158.259	0.014	0.906	0.000683
Snack intake	Diet group × Snack type	1	21	25.433	310.081	1.722	0.204	0.076
Snack intake	Diet group × State	1	21	11.38	158.259	1.51	0.233	0.067
Snack intake	Snack type × State	1	21	2.401	52.341	0.963	0.338	0.044
Snack intake	Diet group × Snack type × State	1	21	2.249	52.341	0.902	0.353	0.041
Wanting	Diet group	1	22	2811.253	33,625.193	1.839	0.189	0.077
Wanting	Snack type	1	22	4788.375	10,061.583	10.47	0.004 *	0.322
Wanting	State	1	22	5914.19	9102.964	14.293	0.001 *	0.394
Wanting	Diet group × Snack type	1	22	106.26	10,061.583	0.232	0.635	0.01
Wanting	Diet group × State	1	22	143.815	9102.964	0.348	0.561	0.016
Wanting	Snack type × State	1	22	0.375	2445.833	0.003	0.954	0.000153
Wanting	Diet group × Snack type × State	1	22	25.01	2445.833	0.225	0.64	0.01
Liking	Diet group	1	22	900.375	34,687.75	0.571	0.458	0.025
Liking	Snack type	1	22	5104.167	12,420.948	9.041	0.006 *	0.291
Liking	State	1	22	658.878	2967.964	4.884	0.038 *	0.182
Liking	Diet group × Snack type	1	22	28.167	12,420.948	0.05	0.825	0.002
Liking	Diet group × State	1	22	208.565	2967.964	1.546	0.227	0.066
Liking	Snack type × State	1	22	158.878	896.755	3.898	0.061	0.151
Liking	Diet group × Snack type × State	1	22	8.461	896.755	0.208	0.653	0.009
Fixation count	Diet group	1	17	219.927	2868.739	1.303	0.269	0.071
Fixation count	Snack type	1	17	91.012	241.506	6.406	0.022 *	0.274
Fixation count	State	1	17	435.285	1726.039	4.287	0.054	0.201
Fixation count	Diet group × Snack type	1	17	30.4	241.506	2.14	0.162	0.112
Fixation count	Diet group × State	1	17	45.93	1726.039	0.452	0.51	0.026
Fixation count	Snack type × State	1	17	3.558	228.256	0.265	0.613	0.015
Fixation count	Diet group × Snack type × State	1	17	0.222	228.256	0.017	0.899	0.000973
Total fixations	Diet group	1	18	0.288	597.079	0.009	0.927	0.000483
Total fixations	Snack type	1	18	19.832	101.453	3.519	0.077	0.164
Total fixations	State	1	18	67.351	125.508	9.659	0.006 *	0.349
Total fixations	Diet group × Snack type	1	18	5.425	101.453	0.963	0.34	0.051
Total fixations	Diet group × State	1	18	0.203	125.508	0.029	0.866	0.002
Total fixations	Snack type × State	1	18	0.344	35.141	0.176	0.679	0.01
Total fixations	Diet group × Snack type × State	1	18	0.000755	35.141	0.000387	0.985	0.0000215

**Table A2:** Output of 3-way (State [hungry or full] **×** Diet group [HFS group or LFS group] × Rating type [wanting or liking] ANOVA model. * *p* < 0.05.

Effect	DFn	DFd	SSn	SSd	F	*p*-Value	Partial Eta Squared
Diet group	1	22	1723.391	31,316.134	1.211	0.283	0.052
State	1	22	2630.273	4825.711	11.991	0.002 *	0.353
Rating type	1	22	5894.584	2840.337	45.657	0.000000863 *	0.675
Diet group × State	1	22	1.5	4825.711	0.007	0.935	0.000311
Diet group × Rating type	1	22	132.423	2840.337	1.026	0.322	0.045
State × Rating type	1	22	656.26	1209.753	11.934	0.002 *	0.352
Diet group × State × Rating type	1	22	174.69	1209.753	3.177	0.088	0.126
